# Co-occurrence of neurofibromatosis type 1 and pseudoachondroplasia – a first case report

**DOI:** 10.1186/s12887-023-03920-7

**Published:** 2023-03-08

**Authors:** Sára Pálla, Pálma Anker, Klára Farkas, Dóra Plázár, Sándor Kiss, Péter Marschalkó, Zsuzsanna Szalai, Judit Bene, Kinga Hadzsiev, Zoltán Maróti, Tibor Kalmár, Márta Medvecz

**Affiliations:** 1 Department of Dermatology, Venereology and Dermatooncology, Semmelweis University, Budapest, 1085 Hungary; 2 Department of Orthopaedics, Semmelweis University, Budapest, 1085 Hungary; 3 Department of Paediatric Orthopaedics, Heim Pál National Children’s Institute, Budapest, 1089 Hungary; 4 Department of Paediatric Dermatology, Heim Pál National Children’s Institute, Budapest, 1089 Hungary; 5 Department of Medical Genetics, Clinical Center, Medical School, University of Pécs, Pécs, 7623 Hungary; 6 Genetic Diagnostic Laboratory, Department of Pediatrics, Faculty of Medicine, Albert Szent-Györgyi Medical School, University of Szeged, Szeged, 6726 Hungary

**Keywords:** Neurofibromatosis 1, Neurofibromin 1, Pseudoachondroplasia, Cartilage oligomeric matrix protein, Case report

## Abstract

**Background:**

Neurofibromatosis type 1 and pseudoachondroplasia are both rare autosomal dominant disorders, caused by pathogenic mutations in *NF1* and *COMP* genes, respectively. Both neurofibromin 1 and cartilage oligomeric matrix protein (COMP) play a role in the development of the skeleton. Carrying both germline mutations has not been previously reported; however, it can affect the developing phenotype.

**Case presentation:**

The index patient, an 8-year-old female presented with several skeletal and dermatologic anomalies resembling the coexistence of multiple syndromes. Her mother had dermatologic symptoms characteristic for neurofibromatosis type 1, and her father presented with distinct skeletal anomalies. NGS-based analysis revealed a heterozygous pathogenic mutation in genes *NF1* and *COMP* in the index patient. A previously unreported heterozygous variant was detected for the *NF1* gene. The sequencing of the *COMP* gene revealed a previously reported, pathogenic heterozygous variant that is responsible for the development of the pseudoachondroplasia phenotype.

**Conclusions:**

Here, we present the case of a young female carrying pathogenic *NF1* and *COMP* mutations, diagnosed with two distinct heritable disorders, neurofibromatosis type 1 and pseudoachondroplasia. The coincidence of two monogenic autosomal dominant disorders is rare and can pose a differential diagnostic challenge. To the best of our knowledge, this is the first reported co-occurrence of these syndromes.

## Background

Neurofibromatosis type 1 (NF1, OMIM: 162200) is a rare autosomal dominant disorder with a birth prevalence of approximately 1 in 2000–3000 individuals [1]. Half of all cases are familial, and 50% are caused by new heterozygous pathogenic mutations in the *NF1* gene (OMIM: 613113). NF1 is a neurocutaneous disorder characterized by café-au-lait macules, axillary and/or inguinal freckling, iris hamartomas, neurofibromas, optic pathway gliomas and distinct bone lesions.

Pseudoachondroplasia (PSACH, OMIM: 177170) was initially described in 1959 and characterized as a distinct skeletal dysplasia separate from achondroplasia [2]. The syndrome is part of the genetically and phenotypically heterogenous osteochondrodysplasia family. PSACH is inherited in an autosomal dominant manner, caused by a heterozygous pathogenic variant in the *COMP* gene (OMIM: 600310) with 100% penetrance, affecting approximately 1 to 9 in 100,000 individuals [3]. Patients with PSACH have normal length at birth followed by the development of disproportionate short-limb short stature usually appearing between the ages of 2 and 4 years. The syndrome is characterized by prominent clinical and radiologic findings on which the diagnosis is based, and genetic testing is required to identify a heterozygous pathogenic variant in *COMP* if clinical features are inconclusive [3–5].

Both neurofibromin 1 and cartilage oligomeric matrix protein, encoded by *NF1* and *COMP* genes, respectively, play a role in the skeletal development [6, 7]. As the co-occurrence of NF1 and PSACH has not been previously described in the literature, there are no data on the effect of both *NF1* and *COMP* mutations on the developing phenotype.

## Case presentation

Here, we report on a young female diagnosed with both NF1 and PSACH and her family. To our knowledge, this is the first case where the presence of these two autosomal dominant disorders explains the phenotype.

**Fig. 1:**
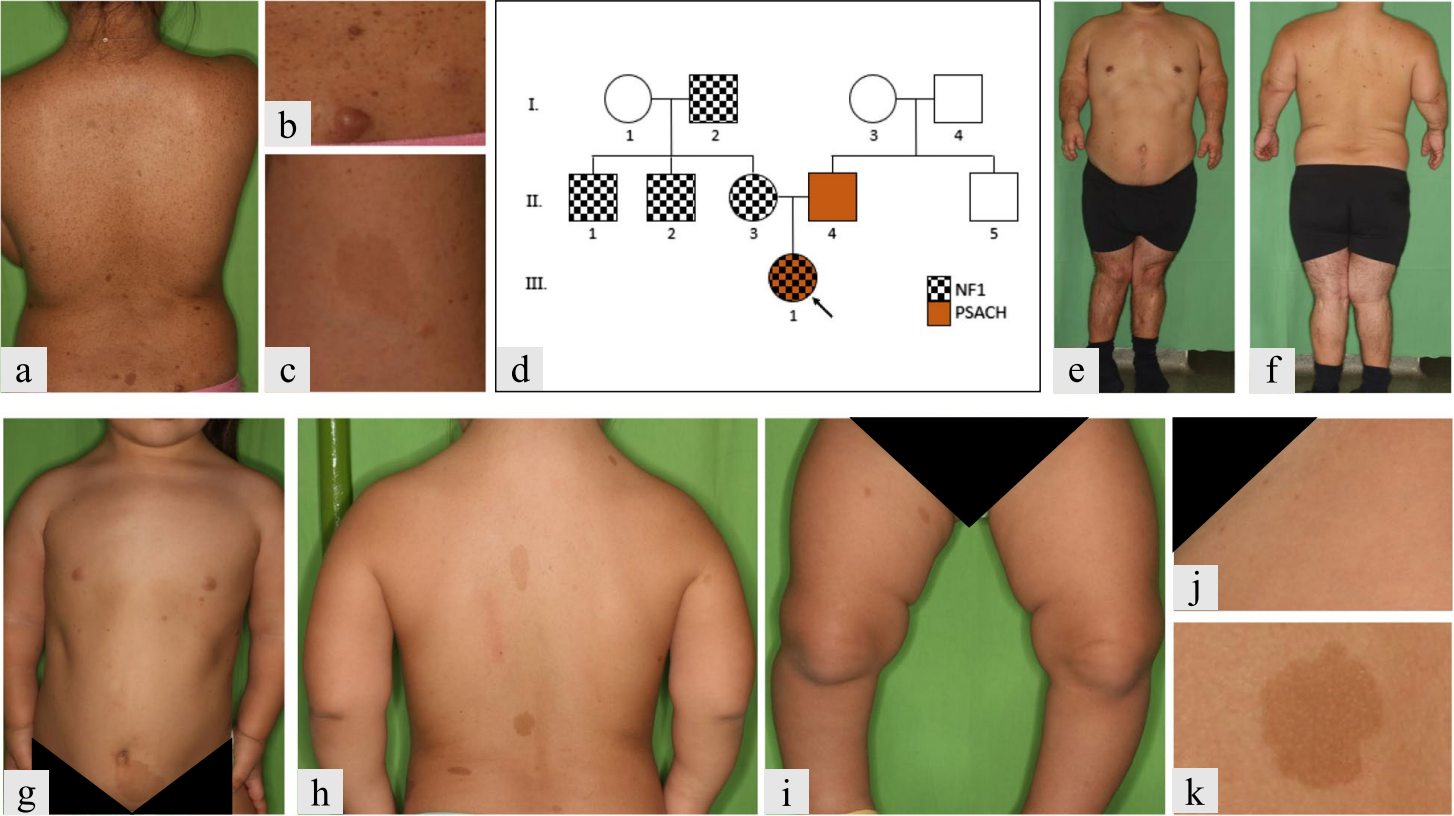
Clinical characteristics of family members and family lineage. The mother presented with clinical characteristics of NF1 (**a**-**c**): cutaneous and subcutaneous neurofibromas (**b**) and CALMs (**c**). The father had a rhizomelic short stature with proximal shortening of the upper limbs (**e**-**f**). Our index patient (**g**-**k**); presented with short stature, proximal shortening of the limbs (**g**-**i**), inguinal freckling (**j**) and multiple CALMs (**h** and **k**). Family lineage shows all affected members in the family (**d**)

An 8-year-old female (III.1) presented to our department with short-limb short stature, waddling gait, genu varum, multiple café-au-lait macules, freckling in the axillary and inguinal region and brachydactyly (Fig. 1g-k). Furthermore, her orthopedic history mentioned lumbar lordosis and knee hyperextensibility. Radiography of the lower limbs had also been performed, which showed axial misalignment of the knee, deformed femoral and tibial epiphyses. The MRI revealed a distinct thickening on the left optic nerve, otherwise her neurologic and ophthalmologic status was negative. Her medical history included a natural birth with average anthropometric parameters (birth weight was 3350 g, length was 50 cm, and head circumference was 33.5 cm); however, her longitudinal growth slowed down with age, and her rhizomelic stature became increasingly apparent, her height was 107 cm (< 0.001 percentile) at 13 years of age.

The mother of the index patient, a 29-year-old female (II.3) presented with inguinal and axillary freckling, several café-au-lait macules (CALMs) larger than 1.5 cm in diameter, and numerous neurofibromas (Fig. 1a-c). Ophthalmologic and neurologic examinations were negative.

The father of the index patient, a 38-year-old male (II.4) presented with disproportionately short stature (height: 147 cm, 0.003 percentile), short limbs, brachydactyly, severe lumbar lordosis, and genu valgum deformity in both lower extremities (Fig. 1e-f). His orthopedic history included severe knee instability on the right side, limited range of motion in the spine, both hips and ankles, severe pes planus on both sides, and severe early osteoarthrosis. Radiography showed vertebral deformities in the lumbar region, and his medical history revealed repeated femoral and tibial corrective osteotomies and lower limb lengthening surgeries.

The molecular analysis of the mother with phenotypical features characteristic for NF1 was performed with a multi-gene panel (QIAGEN QIAseq targeted DNA custom panel kit (Qiagen, Hilden, Germany) on MiSeq device (Illumina, San Diego, USA)) examining *NF1*, *NF2*, *RAF1*, *KIT*, *SPRED1*, *SMARCB1* and *PTPN11* genes. In the *NF1* gene, a previously unreported heterozygous variant c.1479_1480delCTinsG (p.Leu494CysfsTer4) [NM_000267.3] was detected. The NGS-based gene panel sequencing did not detect any disease-causing mutation in genes *NF2*, *RAF1*, *KIT*, *SPRED1*, *SMARCB1* and *PTPN11*.

**Fig. 2:**
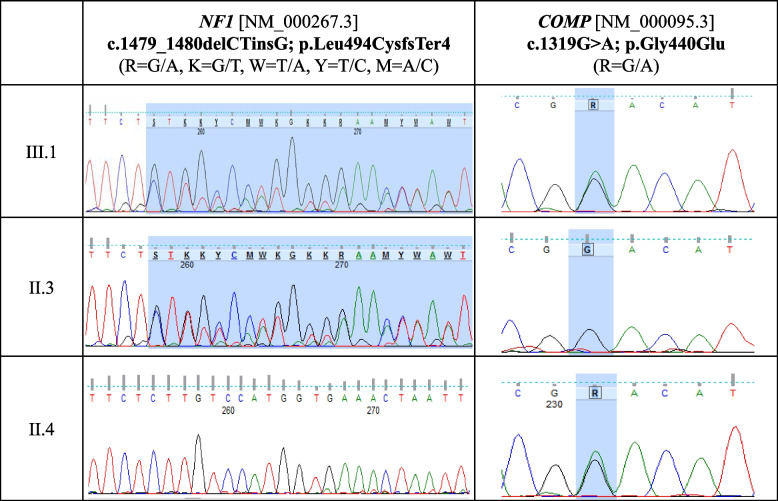
Sequenogram of *NF1* gene exon 13 and *COMP* gene exon 13 of family members. The genetic analysis identified a heterozygous pathogenic variant in the *NF1* (c.1479_1480delCTinsG) and *COMP* (c.1319G > A) genes in the index patient (III.1). The same heterozygous pathogenic variant in the *NF1* and *COMP* gene was detected in the mother (II.3) and in the father (II.4), respectively

The clinical phenotype and family history of the index patient suggested the simultaneous existence of multiple genetically determined disorders. Due to the large number of possible genes associated with the complicated phenotype of the proband, we performed clinical exome sequencing from DNA isolated from peripheral blood with a Trusight One kit on a MiSeq device (Illumina, San Diego, USA). More than 94% of the exons of the examined genes had at least 20-fold coverage, and the average coverage of the investigated genes was between 44.2 and 75.1. The examination detected heterozygous variants in genes *NF1* and *COMP* (Fig. 2). In the *NF1* gene, the same heterozygous variant was identified that was previously detected in the mother [c.1479_1480delCTinsG (p.Leu494CysfsTer4)]. This new variant is likely pathogenic based on mutation analysis softwares (SHIFT, PolyPhen and MutationTaster) and ACMG sequence variant interpretation guidelines, as it is predicted to cause the premature termination of the neurofibromin protein [8–11]. Moreover, the clinical exome sequencing revealed a previously reported pathogenic heterozygous variant in the *COMP* gene [c.1319G > A (p.Gly440Glu)] [NM_000095.3] [12]. All pathogenic variants detected by NGS (gene panel or clinical exome) examination were validated by Sanger sequencing. On the basis of the phenotype and the pathogenic heterozygous variants identified in *NF1* and *COMP* genes, the diagnosis of NF1 and PSACH was established for the index patient (III.1).

In the father, the targeted Sanger sequencing of exon 13 of *NF1* gene and exon 13 of the *COMP* gene was performed, and the same *COMP* variant was identified in a heterozygous form as previously detected in the proband. In the mother, the genetic analysis did not detect any pathogenic variant in *COMP* exon 13.

## Discussion and conclusions

Our case report provided the clinical characteristics and the genetic background of a family with two distinct rare autosomal dominant diseases, and the co-occurrence of these entities in the proband. To the best of our knowledge, this is the first reported case of NF1 associated with PSACH.

Nowadays, the detection of two distinct inherited disorders is becoming more frequent as next-generation sequencing (NGS) techniques are more and more widely available, providing a rapid and inexpensive way to screen for and to diagnose rare syndromes [13, 14]. As the prevalence of NF1 is 1 in 2000-3000, and the prevalence of PSACH in the general population is approximately 1 in 10,000-100,000, the co-occurrence of both syndromes is predicted to occur in 1 in 20,000,000–300,000,000 individuals [1, 3, 14].

NF1 is caused by heterozygous mutations in the *NF1* gene, which, comprising of 60 exons, is one of the largest human genes with one of the highest mutation rates in human genome. Several hot spots with a higher mutation rate such as exons 4b, 7, 10b, 13, 15, 20, 29 and 37 have been described, but no strict genotype/phenotype correlation has been confirmed in large studies [15]. We identified a novel heterozygous germline mutation in the *NF1* gene in exon 13 in the proband and her mother (c.1479_1480delCTinsG), creating a premature translational stop signal (p.Leu494CysfsTer4) in the *NF1* gene. This variant is not present in population databases and has not been reported in the literature in individuals with NF1-related conditions. Loss-of-function variants in *NF1* are known to be pathogenic [16, 17]; thus, this variant is predicted to be pathogenic, explaining the maternal phenotype and the dermatologic characteristics observed in the index patient. Though NF1 can cause short stature, which was present in our index patient [18], the degree of short stature, other orthopedic manifestations, the involvement of the epiphyses and the orthopedic history of the father raised the possibility of the co-occurrence of a separate condition.

PSACH is a rare form of rhizomelic skeletal dysplasia caused by a mutation in the *COMP* gene, where the shortening of the limbs is dominantly proximal, mainly affecting the femur and the humerus. The differential diagnosis includes achondroplasia, multiple epiphyseal dysplasia and mucopolysaccharidosis type IV (also known as Morquio-Brailsford disease) [5, 19]. PSACH families have mostly demonstrated an autosomal dominant inheritance pattern; however, in few cases, spontaneous germline or somatic mutations have been suspected. In few percent, the presence of *COMP* mutation can be due to germline mosaicism affecting the parents; however, limited data are available on this subject [20–22]. In our case, the parents (I.3 and I.4) and the sibling (II.5) of the father were clinically unaffected. A previously reported heterozygous germline mutation in the *COMP* gene was detected in our index patient and her father, explaining the orthopedic manifestations and radiologic findings of these patients. The affected amino acid position is a part of a hydrogen-bonded turn that has a major effect on the overall structure of this protein region, and its disruption can affect the relative positioning of calcium-binding pockets and cause a malfunction of the COMP protein [12, 23]. This variant was initially reported by *Briggs* et al. [12] and was associated with a typical PSACH phenotype observed in our patients as well with short stature, proximal shortening of the limbs, brachydactyly, joint hyperextensibility, waddling gait, and valgum and/or varum deformity of the lower limbs.

Both neurofibromin 1 and COMP proteins play a role in development and growth of the skeleton, and their inactivating mutation can cause several distinct bone deformities [5, 18]. As the presence of simultaneous *NF1* and *COMP* germline pathogenic mutations has not been previously described, no data are available on their effect on the developing phenotype. A comparison of the clinical characteristics of PSACH in the index patient and her father showed that the presence of both *NF1* and *COMP* pathogenic mutations did not significantly aggravate the clinical signs of the skeletal dysplasia in the index patient.

For children diagnosed with NF1, early orthopedic referral and annual examination are suggested [24]. Characteristic, congenital orthopedic manifestations of NF1 include sphenoid wing dysplasia; and tibial dysplasia presenting as anterolateral bowing of the lower leg. Patients with tibial dysplasia can suffer repeated fractures, thus early referral to a pediatric orthopedic surgeon is essential. NF1 can cause dystrophic scoliosis due to vertebral scalloping, penciling of the ribs, spindling of transverse processes and wedging of the vertebral bodies; these features can progress rapidly in childhood requiring surgical intervention [24]. In PSACH, dysplastic changes of the vertebrae include anterior vertebral beaking, persistent oval shape, odontoid dysplasia and platyspondyly, leading to kyphoscoliosis. These vertebral dysplasias, among other orthopedic manifestations such as joint deformities, joint laxity and possible early osteoarthritic changes require regular orthopedic examinations [5, 19]. Hence, both NF1 and PSACH can cause major skeletal deformities, a close orthopedic follow-up is required, and the orthopedic status of our proband should be re-evaluated at least yearly.

Both NF1 and PSACH affect the stature and growth rate of patients. Thirteen percent of patients with NF1 have a height ≥ 2 standard deviations below the population mean (< 160 cm for males and < 147 cm for females), whereas the height of adult PSACH patients usually ranges between 82 and 130 cm [19, 25]. Growth curves are available for children with NF1 to help the clinician determine whether the height of the patient is consistent with the diagnosis. If the degree of short stature is more severe, it suggests an additional cause, and further evaluation is recommended [26].

For parents, genetic counselling and planned pregnancy are of most importance, considering that the risk of passing down the pathogenic mutation is 50% for a single gene, and the risk for the co-occurrence of both syndromes in their next child is 25%.

Here, we described the first coincidence of two autosomal dominant syndromes, NF1 and PSACH. The co-occurrence of two monogenic disorders is a rare phenomenon and posed a differential diagnostic challenge despite the fact that all observed clinical features were typical for NF1 or PSACH. With this case report, we emphasize that when a monogenic disease is diagnosed with distinct phenotypic features that cannot be explained by the syndrome, the presence of a separate disease should be considered, and an extended molecular examination should be performed.

## Data Availability

Not applicable.

## Abbreviations

NF1: Neurofibromatosis type 1
NGS: Next-generation sequencing
PSACH: Pseudoachondroplasia
OMIM: Online Mendelian Inheritance in Man
COMP: Cartilage Oligomeric Matrix Protein
CALMs: Café-au-lait macules
